# Case Report: A Retrospective Serological Analysis Indicating Human Exposure to Tick-Borne Relapsing Fever Spirochetes in Texas

**DOI:** 10.1371/journal.pntd.0003617

**Published:** 2015-04-09

**Authors:** Hannah K. Wilder, Edward Wozniak, Elizabeth Huddleston, Sri Ramya Tata, Nicholas C. Fitzkee, Job E. Lopez

**Affiliations:** 1 Department of Pediatrics, Section of Tropical Medicine, Baylor College of Medicine and Texas Children’s Hospital, Houston, Texas, United States of America; 2 Texas State Guard, Medical Brigade, Uvalde, Texas, United States of America; 3 Department of Biological Sciences, Mississippi State University, Starkville, Mississippi, United States of America; 4 Department of Chemistry, Mississippi State University, Starkville, Mississippi, United States of America; University of California San Diego School of Medicine, UNITED STATES

## Presentation of Case

In June 2013, a Caucasian male from Kerr county, Texas, with an extensive history of outdoor activity working with sheep, goats, and exotic game became ill, displaying fever, chills, uveitis, headache, retrobulbar pain, severe malaise, and weakness. Myalgia was centered on upper extremities, most notably the shoulders, arms, and hands, and by July 2013 the patient had experienced two febrile episodes (39°C). Numerous insect bites were reported, most notably on areas of the body that were in direct contact with the ground, but offending arthropods were not found. The patient was initially tested for *Coxiella burnetti* exposure, but repeated serological results demonstrated that phase I and phase II immunoglobulin G (IgG) antibody titers failed to increase. Antibiotic treatment was administered, and symptoms improved. A retrospective evaluation of the patient’s history and clinical summary led to suspicion of tick-borne relapsing fever borreliosis. The patient did not have a history of traveling outside of Texas prior to the onset of symptoms, and *Borrelia turicatae* was suspected as the causative agent. A serum sample was sent to us four months after antibiotic treatment, and we implemented a molecular approach to determine seroreactivity against two diagnostic antigens for relapsing fever spirochetes, recombinant glycerophosphodiester phosphodiesterase (rGlpQ) and *Borrelia* immunogenic protein A (rBipA).

## Approach

### Ethics Statement

Verbal and written informed consent was obtained from the patient to test the serum sample. The study was submitted to the Mississippi State University Institutional Review Board, protocol #13–369, and approved by designated review.

### Protein Analysis, Production of Diagnostic Antigens, and Serological Testing

rGlpQ has been used to evaluate mammalian exposure to species distributed in the western United States and East and West Africa [1–4]. The gene is absent from other pathogenic spirochetes, and Schwan and colleagues first characterized rGlpQ as an antigen that could discriminate between exposure to relapsing fever and Lyme-causing *Borrelia* [1]. Amino acid alignments of GlpQ using Vector NTI 11.5 software (Life Technologies, Foster City, California, US) indicated the *B*. *turicatae* homologue is highly conserved between New (*B*. *turicatae*, *Borrelia parkeri*, and *Borrelia hermsii*) and Old (*Borrelia recurrentis*) World species of relapsing fever spirochetes (Table 1).

**Table 1:** Percent amino acid identity of GlpQ between species of relapsing fever spirochete.

	*B*. *turicatae*	*B*. *parkeri*	*B*. *hermsii*	*B*. *recurrentis*
*B*. *turicatae*	-			
*B*. *parkeri*	97	-		
*B*. *hermsii*	87	87	-	
*B*. *recurrentis*	81	80	80	-

To produce *B*. *turicatae* GlpQ as a recombinant fusion protein, the gene was amplified using genomic DNA from the 91E135 isolate with 5ʹ-CACCATGAAATTAATTAAAACAA AATTATTAAT GCTTACAATGAATATTTTT-3ʹ and 5ʹ-TTGTTTTACAAACTTCACTAC TGTATCA GTAAAATCTGTAAAT-3ʹ forward and reverse primers, respectively. The amplicon was cloned into the pET102 expression vector, and the absence of nucleotide errors was confirmed by sequencing analysis using Vector NTI Advanced 11.5 software (Life Technologies). rGlpQ was produced as a six-histidine and thioredoxin fusion protein and purified by immobilized metal affinity chromatography using HisTrap FF Crude columns precharged with Ni^2+^ (GE Healthcare Life Sciences, Pittsburgh, Pennsylvania, US). Thioredoxin was removed by incubating the purified protein with 0.01 U of EKMax Enterokinase (Life Technologies), following the manufacturer’s protocol. The molecular mass of native GlpQ and rGlpQ are 40 and 45 kDa, respectively.

Previous studies reported the identification and antigenicity of BipA and indicated a homologue was absent outside of relapsing fever *Borrelia* [5,6]. BipA also shares 24%–76% amino acid identity between relapsing fever spirochete homologues (Table 2) and was demonstrated to differentiate between infections caused by *B*. *turicatae* and *B*. *hermsii* [5]. *B*. *turicatae* rBipA was produced using the pET102 expression system as previously described [5], and thioredoxin was left attached to maintain protein solubility. The molecular mass of native and rBipA are 60 and 75 kDa, respectively.

**Table 2:** Percent amino acid identity of BipA between species of relapsing fever spirochete.

	*B*. *turicatae*	*B*. *parkeri*	*B*. *hermsii*	*B*. *recurrentis*
*B*. *turicatae*	-			
*B*. *parkeri*	76	-		
*B*. *hermsii*	36	34	-	
*B*. *recurrentis*	25	24	26	-

**Fig 1:**
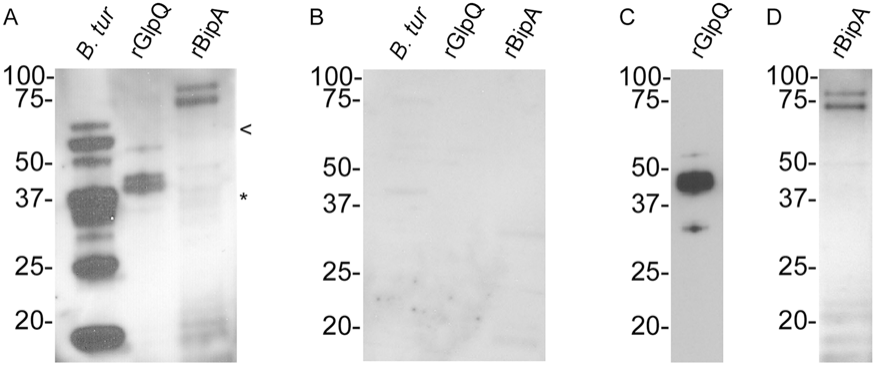
Immunoblotting to evaluate antibody binding to *B*. *turicatae* protein lysates, rGlpQ, and rBipA (A). The asterisk and arrowhead represent the molecular masses for native GlpQ and BipA, respectively (A). rBipA was produced as a thioredoxin fusion protein and is 15 kDa larger than the native protein. Immunoblots were also probed with a serum sample from an uninfected patient (B) and a monoclonal antibody against the six-histidine fusion tag (C and D). Molecular masses are shown to the left of each immunoblot.

Protein lysates from 1 x 10^7^ spirochetes and 1 μg of rGlpQ and rBipA were electrophoretically separated and transferred to nitrocellulose membranes as previously described [5] using TGX gels, Mini-PROTEAN Tetra cell, and the Mini Trans Blot system (BioRad, Hercules, California, US). Immunoblots were probed with the patient’s serum sample at a 1:400 dilution, and Rec-Protein G-HRP (Life Technologies) diluted 1:4,000 was used as the secondary molecule. Serological reactivity to multiple proteins in the *B*. *turicatae* whole spirochete lysates, rGlpQ, and rBipA (Fig. 1A) was detected by chemiluminescence and indicated exposure to relapsing fever spirochetes. A serum sample from a subject living in a nonendemic region of the US without a history of exposure to relapsing fever spirochetes was used as a negative control (Fig. 1B). The nitrocellulose membrane was subsequently probed with a Monoclonal Anti-polyHistidine-Peroxidase antibody (Sigma-Aldrich, St. Louis, Missouri, US), indicating the presence of the recombinant proteins (Fig. 1C and D). Additionally, the patient’s antibody titers to rGlpQ and rBipA were over a 1:6,400 dilution.

## Case Discussion

The ecological overlap and nonspecific symptoms caused by vector-borne bacterial, viral, and parasitic pathogens signifies the importance of utilizing improved molecular assays to accurately determine pathogen exposure. In this report, the patient’s extensive outdoor activity and nonspecific clinical manifestation made it difficult to initially determine the causative agent. Retrospective analysis of serum reactivity to rGlpQ and rBipA suggested likely exposure to *B*. *turicatae*. A potential limitation of BipA as a species-specific antigen is that *B*. *turicatae* and *B*. *parkeri* are closely related [7], and the homologues share 76% amino-acid identity. However, we are unaware of a report indicating the occurrence of *Ornithodoros parkeri*, the vector for *B*. *parkeri*, in Texas, as the ticks have been collected throughout the western and midwestern US [8,9], further causing us to suspect *B*. *turicatae*.

Accurate epidemiological studies for tick-borne relapsing fever borreliosis in Texas have been challenging because of nonspecific symptoms and limited molecular diagnostic assays. Human exposure has been evaluated retrospectively, by confirming spirochete colonization of the tick vector, *Ornithodoros turicata*, collected from presumed contact sites, which frequently included caves and manmade dugouts [10,11]. The disease has also been misdiagnosed as Lyme borreliosis because of similar neurological symptoms [10]. Further complicating a correct diagnosis was serological cross-reactivity with immunofluorescent assays (IFA) and enzyme-linked immunosorbent assays (ELISA), in which patient serum samples were assessed for reactivity to fixed *Borrelia burgdorferi* or total protein lysates, respectively [10]. Given the number of conserved antigens between *Borrelia* species and observed serological cross-reactivity [12,13], IFA and ELISA may be misleading. Furthermore, Ixodid ticks that transmit *B*. *burgdorferi* and feed for 5–7 days were not found to be attached on the patients [10].

Transmission of *B*. *turicatae* from the tick and the ecology and feeding behavior of *O*. *turicata* indicate that outdoor enthusiasts, military ground personnel, and low-income families living in primitive housing conditions are at-risk populations [14,15]. The ticks complete their blood meal within 5–60 minutes and subsequently return to the cave crevice, nest, or den in which they cohabit with small mammals [10,16]. Consequently, *O*. *turicata* is rarely found on the host, and a full blood meal is not required for transmission and infection [14]. Moreover, mammalian hosts supporting the maintenance of *B*. *turicatae* in nature are not completely known. Schwan and colleagues isolated the spirochetes from symptomatic domestic dogs, while the tick vector has been collected in caves [10,17,18]. These findings suggest a role of wild canids and bats in *B*. *turicatae* maintenance. Our ecological studies in Texas utilizing rGlpQ and rBipA to determine small mammal exposure to *B*. *turicatae* indicate coyotes and rodents may maintain the pathogens (manuscript in preparation). As improved molecular assays are utilized to evaluate mammalian exposure to relapsing fever spirochetes, we will further define the ecology and human health burden in regions where the pathogens are overlooked.

**Key Learning Points**

- Given nonspecific clinical symptoms, relapsing fever spirochetes are likely underdiagnosed.
- Serological responses to rGlpQ and rBipA can indicate exposure to relapsing fever spirochetes.
- Likely at-risk populations include outdoor enthusiasts, military ground personnel, and those living in primitive housing conditions.
